# Inducible Nitric Oxide Synthase/Nitric Oxide System as a Biomarker for Stress and Ease Response in Fish: Implication on Na^+^ Homeostasis During Hypoxia

**DOI:** 10.3389/fphys.2022.821300

**Published:** 2022-05-17

**Authors:** M. C. Subhash Peter, R. Gayathry, Valsa S. Peter

**Affiliations:** ^1^ Inter-University Centre for Evolutionary and Integrative Biology iCEIB, School of Life Science, University of Kerala, Kariavattom, Thiruvananthapuram, India; ^2^ Department of Zoology, University of Kerala, Kariavattom, Thiruvananthapuram, India

**Keywords:** fish, stress response, ease response, nitric oxide, NOS, hypoxia, Na^+^/K^+^-ATPase, biomarker

## Abstract

The cellular and organismal response to stressor-driven stimuli evokes stress response in vertebrates including fishes. Fishes have evolved varied patterns of stress response, including ionosmotic stress response, due to their sensitivity to both intrinsic and extrinsic stimuli. Fishes that experience hypoxia, a detrimental stressor that imposes systemic and cellular stress response, can evoke disturbed ion homeostasis. In addition, like other vertebrates, fishes have also developed mechanisms to recover from the impact of stress by way of shifting stress response into ease response that could reduce the magnitude of stress response with the aid of certain neuroendocrine signals. Nitric oxide (NO) has been identified as a potent molecule that attenuates the impact of ionosmotic stress response in fish, particularly during hypoxia stress. Limited information is, however, available on this important aspect of ion transport physiology that contributes to the mechanistic understanding of survival during environmental challenges. The present review, thus, discusses the role of NO in Na^+^ homeostasis in fish particularly in stressed conditions. Isoforms of nitric oxide synthase (NOS) are essential for the synthesis and availability of NO at the cellular level. The NOS/NO system, thus, appears as a unique molecular drive that performs both regulatory and integrative mechanisms of control within and across varied fish ionocytes. The activation of the inducible NOS (iNOS)/NO system during hypoxia stress and its action on the dynamics of Na^+^/K^+^-ATPase, an active Na^+^ transporter in fish ionocytes, reveal that the iNOS/NO system controls cellular and systemic Na^+^ transport in stressed fish. In addition, the higher sensitivity of iNOS to varied physical stressors in fishes and the ability of NO to lower the magnitude of ionosmotic stress in hypoxemic fish clearly put forth NO as an ease-promoting signal molecule in fishes. This further points to the signature role of the iNOS/NO system as a biomarker for stress and ease response in the cycle of adaptive response in fish.

## Introduction

Stress is a condition of threatened or disturbed physiological homeostasis when animals perceive stressor-driven stimuli of extrinsic or intrinsic origin. As a result, when confronted with stressors, vertebrates show a stress response, that is, an integrated set of physiological and behavioral actions coordinated by the brain and directed at the survival of the threatened individual. Fishes are very sensitive to stressors. Much more than in terrestrial animals, the ionosmotic equilibrium in fish is disturbed by stressors Chrousos and Gold (1992); Wendelaar Bonga (1997); Iwama et al. (2006); Peter (2011); Peter and Peter (2011); Islam et al. (2020); Cerqueira et al. (2021). Teleost fishes have evolved strategies to maintain ion homeostasis, irrespective of the osmolarity and ionic concentrations of the water. In freshwater, the supply of ions is restricted and fish gain water osmotically and lose ions passively. For compensation, the fish take up ions from the water *via* gills with excretion of copious urine and with minimal drinking (Verbost et al., 1994; Marshall and Grosell, 2006; Guh et al., 2015). Seawater fishes while facing osmotic water loss and passive influx of ions drink actively and secrete excess of Na^+^ and Cl^−^ (Karnaky, 1998; Iwama et al., 2006; Islam et al., 2020). Euryhaline fishes that live in the water of varying osmolarity or migrate between freshwater and seawater possess a remarkable ability to either upregulate or downregulate their ion regulatory mechanisms to retain osmotic and ionic constancy in their internal fluid compartments (Selvam et al., 2014; Dutta et al., 2019; Kumar et al., 2020). Thus, regulation of the ion balance is vital to fish and demands the active participation of the gills, kidneys, and intestines (Marshall and Grosell, 2006; Peter, 2007; Peter et al., 2011; Peter et al., 2011).

Fishes tolerate hypoxia by exerting a set of concerted physiological responses (Fago and Jensen, 2015; Gattuso et al., 2018). Limited O_2_ availability in the environment or in internal tissues demands decrease of O_2_ consumption rates, protection against oxidative damage, and redistribution of blood flow into circulation, at least in air-breathing species (Gattuso et al., 2018). All these responses require the activation of a complex network of intracellular cascades, such as those related to nitric oxide (NO) and its metabolites, nitrite and nitrate, to control and coordinate the molecular circuits that sustain adaptive hypoxia-dependent physiological responses (Fago and Jensen, 2015). In fishes, a critical role of NO and its metabolites, in particular, nitrite and S-nitrosothiols has been recognized as key regulators under hypoxic challenges as in nonmammalian species (Gattuso et al., 2018).

Fishes that experience hypoxia impose systemic and cellular stress response and evoke disturbed ion homeostasis. This ionosmotic stress response demands modulation in the functional attributes of ion transporters, including Na^+^/K^+^-ATPase (NKA). To achieve tolerance to stress, like other vertebrates, fishes have also developed mechanisms to recover from the impact of stress by way of shifting stress response into ease response that could attenuate the magnitude of stress response with the aid of certain neuroendocrine signals. As a potent molecule that can protect hypoxia stress, NO can also attenuate the impact of hypoxia-induced ionosmotic stress response in fish. However, limited information is available on the role of NO in the cellular and organismal response to ionosmotic stress response in fishes. The present review, thus, discusses the role of NO in the regulation of Na^+^ homeostasis in fish, particularly in stressed conditions. The higher sensitivity of NO to varied physical stressors in fishes clearly puts forth NO as an ease-promoting signal molecule in fishes. As a result, a critical role of NO in stress and ease response in the cycle of adaptive response has been identified in fish. It is envisaged that the integrative action of NO in ion transport physiology would further contribute to the mechanistic understanding of the survival of fishes during hypoxia.

### The Stress Response in Fish

The concept of stress has been widely accepted by biologists ever since it was first described by Selye (1936) as a general reaction of mammals to a diversity of stimuli experienced as noxious or threatening. The concept has now been extended to almost all vertebrate species. At the organismal level, stress can be defined as a condition of disturbed physiological homeostasis due to the action of intrinsic or extrinsic stimuli, generally recognized as stressors (Chrousos and Gold, 1992; Wendelaar Bonga, 1997). Fish experience stressors under natural and aquaculture conditions. For example, management practices such as grading, confinement, and air exposure of fishes induce stress (Barton and Iwama, 1991). In addition, toxic water pollutants besides their direct toxic effects evoke physiological disturbances in fish, resulting in a stress response (Wendelaar Bonga, 1997; Iwama et al., 2006). Compensatory actions are, therefore, necessary for fish to accommodate the effects of stressful stimuli.

A complex network of neuroendocrine hormones interacts with physiological processes and exhibits adaptive response in the event of exposure to these stressors of biotic or abiotic origin. Fishes have evolved various physiological and compensatory mechanisms that produce simple or integrated adaptive stress responses that are expressed at all levels of biological organization (Wendelaar Bonga, 1997; Dini et al., 2006; Iwama et al., 2006; Peter, 2007; Peter et al., 2011). Furthermore, these stressors that induce stress stimuli disturb all physiological processes, including acid/base, osmotic, and metabolic regulation (Peter and Peter, 2011; Islam et al., 2020). Consequently, stress stimuli lead to production of biochemical and physiological stress responses that could extend at the primary, secondary, and tertiary levels (Barton, 2002; Iwama et al., 2006). As efficient chemical signals, neurohormones and hormones released from endocrine axes coordinate almost all physiological processes and initiate their actions to maintain cellular and systemic homeostasis. In fishes, major endocrine stress axes compress brain–sympathetic–chromaffin (BSC) and hypothalamo–pituitary–interrenal (HPI) axes that produce catecholamines and corticosteroids as stress hormones and lead major roles in evoking stress response in fish (Barton and Iwama, 1991; Sumpter, 1997; Wendelaar Bonga, 1997; Iwama et al., 2006). A stress stimulus activates sympathetic nerve fibers that innervate chromaffin cells located in the anterior head kidney (Reid et al., 1998). Chromaffin cells, in turn, release catecholamines mainly as epinephrine *via* cholinergic receptors into the circulation, and its levels increase immediately upon stress (Randall and Perry, 1992; Reid et al., 1998). The release of cortisol from the HPI axis generally takes more time than catecholamine release. Activation of CRH, or CRF, chiefly from the hypothalamus in the brain drives corticotrophic cells of the anterior pituitary to secrete adrenocorticotropin (ACTH) that stimulates the interrenal cells in the kidneys to synthesize and release cortisol into the blood (Barton and Iwama, 1991; Wendelaar Bonga, 1997).

In all vertebrate groups, catecholamines are released into the general circulation under conditions that require enhanced blood oxygen transport and mobilization of energy substrates (Mazeaud et al., 1997). Therefore, the release of catecholamines is an integral part of the physiological response to stressors in all vertebrate groups (Hart et al*.,* 1989; Wendelaar Bonga, 1997; Barton, 2002). The stress response in fish includes a marked increase in the oxygen uptake rate of the gills as a result of increased ventilation rate, stimulated branchial blood flow and branchial oxygen diffusing capacity, and increased oxygen transport capacity of the blood (Perry, 1997). The stress-related hyperglycemia reported in many species of teleosts (Barton and Iwama, 1991; Iwama et al., 2006; Peter, 2011) is mediated mainly by the effects of catecholamines on glucose release from the liver. The high peak levels of catecholamines, particularly adrenaline during acute stress, are held responsible for the increase in permeability to water and ions of the gills (Mazeaud et al., 1997). It may also cause disruption of the structure of the gill lamellae, including epithelial lifting (Wendelaar Bonga, 1997). The high circulating catecholamine levels during chronic stress may lead to desensitization of the regulatory or target cells, mainly through the downregulation of receptors on these cells (Wendelaar Bonga, 1997). Furthermore, the acute and chronic catecholamine levels can cause damage to the gills, resulting in disturbance of ion regulation and other functions of the gills (Mazeaud et al., 1997).

The HPI axis releases cortisol in response to stressful stimuli (Donaldson, 1981; Wendellar Bonga, 1997; Peter and Peter, 2011). This steroid is released in response to various hormones, of which the adreno-corticotropic hormone from the pituitary gland is most important. The release of the adrenocorticotropic hormone is under the control of the corticotropin-releasing hormone of the hypothalamus. An elevation of plasma cortisol, which can be reliably determined, is most widely used as an indicator of stress in fish. Consequently, the role of cortisol in hydromineral regulation performance in stressed fishes has been the subject of many studies (Barton and Iwama, 1990; Sumpter, 1997; Seidelin and Madsen, 1999; Mancera and McCormick, 1999; Nolan et al., 1999; Dang et al., 2000; McCormick, 2001; Metz et al., 2003; Peter and Peter, 2011). Cortisol exerts a broad-activity spectrum in fishes having both mineralocorticoid and glucocorticoid functions (Vijayan et al., 2003; Iwama et al., 2006). Similar to freshwater or seawater adaptation, in stressed fish, cortisol is important for the proliferation of CCs and promotes the synthesis of Na^+^, K^+^-ATPase activities in these cells (Dang et al., 2000; Peter et al., 2011). The very high circulating levels of cortisol in stressed fish have also been shown to promote apoptosis of the chloride cells in the gills (Bury et al., 1998).

### The Ease Response in Fish

**FIGURE 1:**
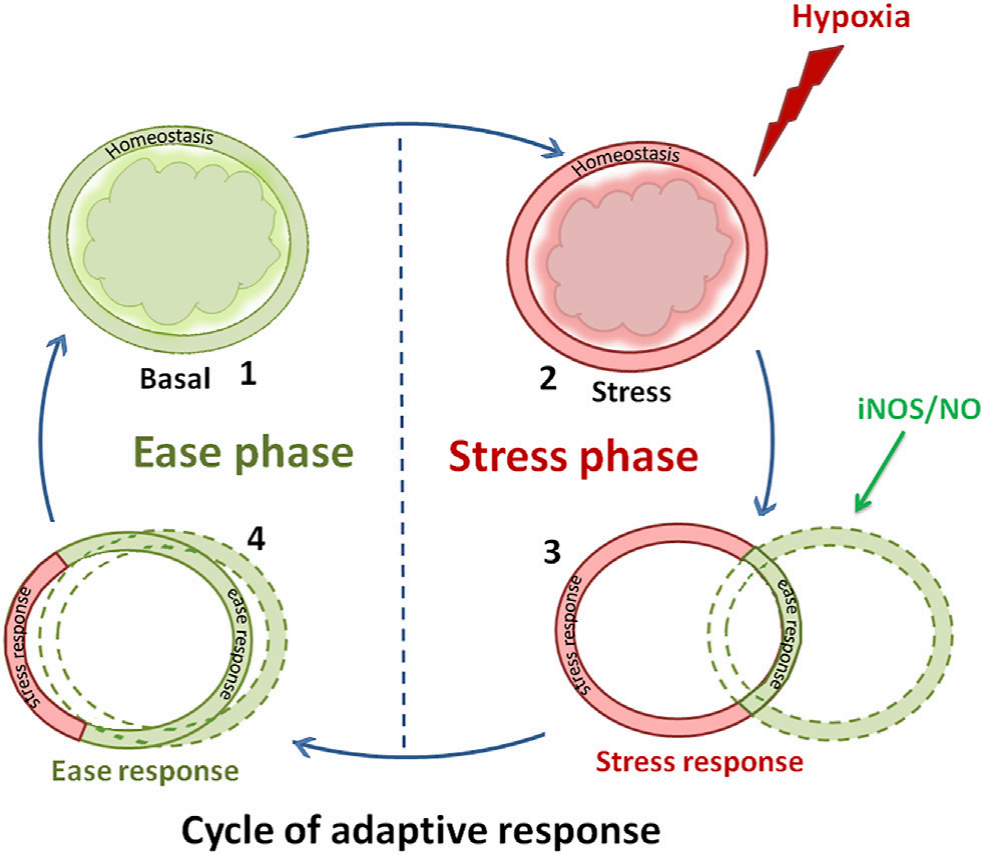
Schematic chart showing the cyclic events associated with the adaptive response that happens in an organism when it encounters stressors such as hypoxia. In this cycle of adaptive response, the organism exhibits a basal homeostatic state due to its nonstressed condition (Panel 1.1). But later, the organism perceives stress stimulus from hypoxia. For example, when an air-breathing fish was held in water, it experienced hypoxia as a result of forceful submergence (immersion) (Panel 1.2). Here, the fish enters the stress phase and shows stress response (Panel 1.2). When these fish were treated with L-NAME, activation of the iNOS/NO system occurs in their ionocytes that induces multidimensional regulation of NKA. This brings a new drive to initiate ease response in this fish, though its contribution is little at that stage of the stress phase (Panel 1.2). However, in the next stage, the fish were able to utilize the fullest iNOS/NO system to lower the magnitude of the stress response as evident in the overlapping of the highest ring of ease response (Panel 1.3). In stage 3, the fish were able to show a substantial degree of ease response, though minimal stress response could also be sustained in that stage (Panel 1.4). While staying in this phase of ease, the fish could be brought back to its basal homeostasis stage (Panel 1.1). At this basal homeostatic stage, the fish could again experience another stress stimulus and corresponding stages could be achieved repeatedly by running the events of the cycle again. This constitutes the cycle of adaptive response with two phases, namely, stress and ease phase (Panel 1).

There occurs an urge in all animals to nullify the impact of the physiological and behavioral disturbances when they develop stress response upon stressor exposure. The response of fish to a stressor that imposes an allostatic load on physiological homeostasis further demands correction of disturbed homeostatic control and recovery action utilizing physiological machineries. As a complex system that works for correcting physiological disturbances, fishes rely on integrative and compensatory physiological modifications (Peter and Peter, 2011). At the onset of stressor perception and subsequent stress response, fishes tend to recover or regain disturbed homeostatic control. They develop adaptive response directed toward accommodating the imposed stressor through the process of stress-acclimation that evokes stress response (Peter, 2013a). Similarly, with the appropriate direction of neuroendocrine signals such as serotonin and melatonin, a phase of recovery happens as an adaptive response during the process of acclimation, wherein the animal recovers from the impact of stress response (Peter, 2013a). During this phase of adaptive response, with the support of a physiological network, the animal could reduce the magnitude of its disturbed stress response to the basal homeostatic state (Peter, 2013a). This state of ease and ease response, thus, can reduce the allostatic load with the combined effort of a specific neuroendocrine signal or cytogenic ligand such as NO along with the physiological processes including ionosmotic regulation (Peter, 2013a). Stress and ease responses, thus, appear as the innate mechanisms that work together as the cycle of adaptive response in vertebrates that holds both stress and ease phases (Figure 1).

Sensitization/desensitization of hormonal receptor systems appears to be one of the target processes that direct ease response (Peter, 2013a). It is well-known that fishes rely on many hormonal and chemical signals that target almost all physiological processes to correct their disturbed physiological homeostasis upon stressor exposure. During this process of adaptive response, a phenomenon of inter-hormonal interference could operate as a mechanism of hormonal interaction during stress and post-stress acclimation (Peter and Peter, 2011). For example, THs and cortisol have been shown to produce interactive actions in these adaptive phases that would enhance the survival capacity of fish to tolerate stress-induced allostatic load, including ambient salinity exposure (Peter et al., 2011; Peter and Peter, 2011) or hypoxia (Peter and Simi, 2017). Now it appears that to drive ease response, fish could rely on cytogenic signaling molecules such as NO which could ultimately direct the ease response in the fish, favoring them to achieve a successful acquisition of adaptive response to counteract stress (Figure 1).

## Nitric Oxide

**FIGURE 2:**
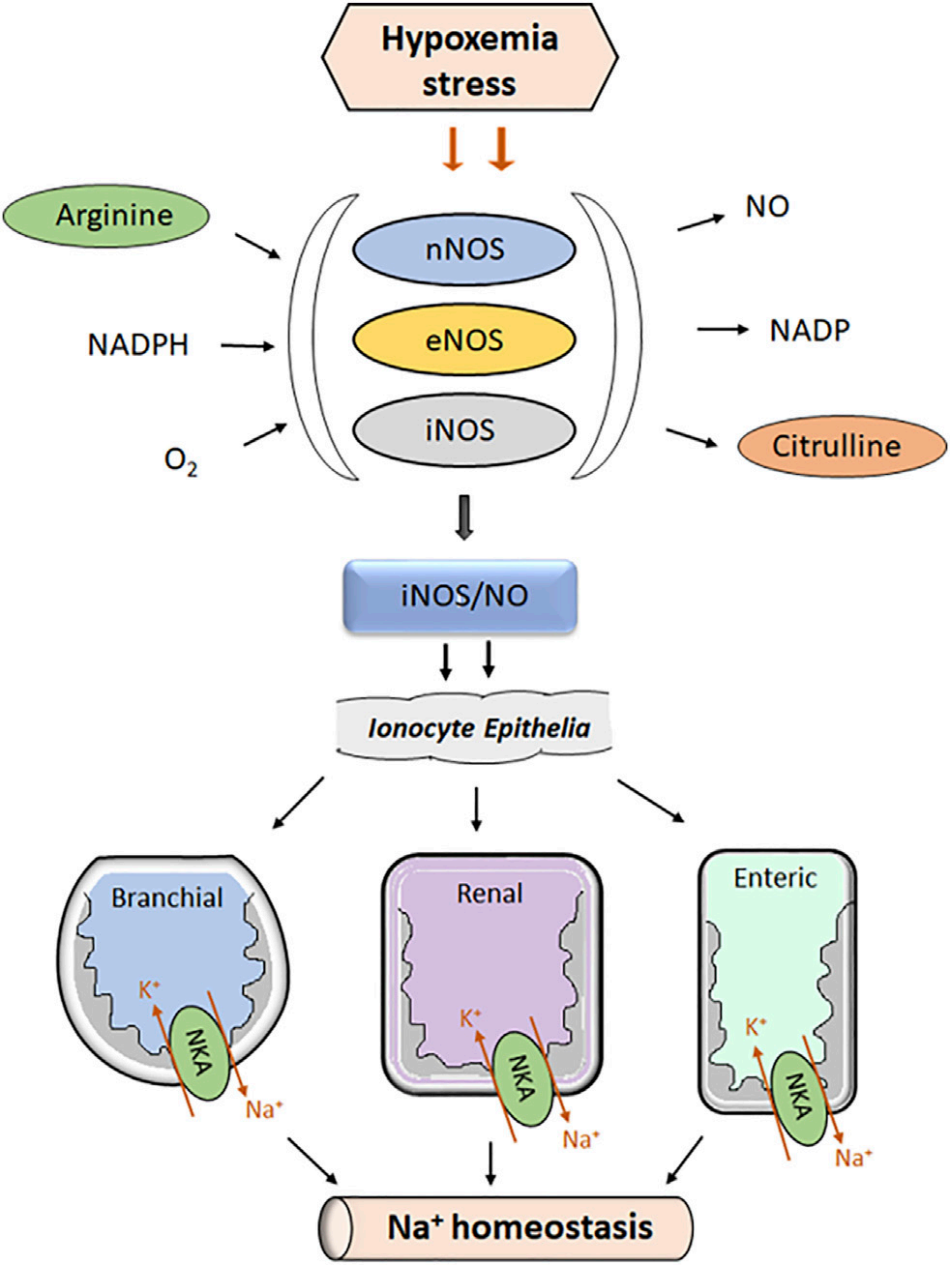
Chart showing the impact of hypoxemia stress on the activation of the iNOS/NO system in the ionocytes of fish. In fishes, varied isoforms of nitric oxide synthases: endothelial (eNOS), neuronal (nNOS), and inducible (iNOS) are involved in the production of NO from L-arginine in the presence of NADPH and O_2_. Upon induction of hypoxemia stress in an air-breathing fish (*A. testudineus*), activation of the iNOS/NO system occurs in branchial ionocytes. This leads to the differential regulation of NKA that ultimately contributes to the recovery of systemic Na^+^ balance.

Nitric oxide is an endogenously synthesized signaling molecule that plays a multitude of physiological actions in organ systems of vertebrates, including fishes. As a highly diffusible gas with a short half-life of a few seconds, NO permeates through all biological membranes and mediates both autocrine and paracrine actions (Gally et al., 1990; Snyder and Bredt, 1992). Although the nonenzymatic pathways of NO synthesis occur, endogenous NO is derived largely from the enzymatic pathways. A series of redox reactions starting from the degradation of L-arginine to L-citrulline in the presence of oxygen and NADPH that act as substrates lead to the formation of NO (Moncada and Higgs, 1993; Wu and Morris, 1998; Chen and Popel, 2009). Varied isoforms of nitric oxide synthases (NOS) are involved in the synthesis of NO (Figure 2). Three isoforms of NOS, namely, neuronal NOS (nNOS), inducible NOS (iNOS), and endothelial NOS (eNOS) exist with different catalytic properties and inhibitor sensitivity. nNOS and eNOS are constitutive enzymes that are controlled by intracellular Ca^2+^/calmodulin, whereas iNOS is inducible at the level of gene transcription and Ca^2+^ independence in target cells (Althaus, 2012). In addition, there is evidence of the production of NO from nitrite *via* the nonenzymatic pathways, especially under acidic conditions (Zweier et al., 1999; Li et al., 2008). Generally, iNOS expression is limited in cells (Cho et al., 1992; Ruan et al., 1996) but induced by the products of inflammatory cytokines such as tumor necrosis factor (Vallance and Moncada, 1994), bacterial endotoxins (Hibbs et al., 1988), and exotoxins (Zembowicz and Vane, 1992). iNOS can produce a large amount of NO in nanomoles over a long period of time (hours/days) once it is expressed, though its high concentrations can have toxic effects (Hibbs et al., 1988; Griffith and Stuehr, 1995; Kröncke et al., 1997).

### Actions of Nitric Oxide

As one of the oldest signaling molecules that act as both intercellular and intracellular messengers, NO participates in diverse physiological functions in mammals, including vasodilation, smooth muscle relaxation, neurotransmission, and immune response (Althaus, 2012). nNOS is primarily found in the nervous system and is necessary for neuronal signaling. eNOS is localized to the endothelium and is essential for vasodilation and control of blood pressure, producing nanomolar amounts of NO for short periods in a calcium-/calmodulin-dependent manner. In contrast, iNOS is not constantly present in cells and is only expressed when the cell is induced or stimulated, typically by proinflammatory cytokines and/or bacterial lipopolysaccharide (LPS) (Choudhury and Saha, 2016). The iNOS/NO system that is present in a variety of tissues including epithelia and macrophages are independent of Ca^2+^ and released in large amounts for long periods against endotoxin and cytotoxic exposure (Griffith and Stuehr, 1995; Napoli and Ignarro, 2003; Choudhury and Saha, 2012a; Choudhury and Saha, 2012b; Kumari et al., 2018; Hasan et al., 2020; Das et al., 2021). The members of NOS drive inter and intracellular signaling in target tissues and produce systemic and physiological actions in almost all organ systems.

NO activates guanylate cyclase and elevates cGMP levels, resulting in relaxation and vasodilation (Ignarro, 1989), involving cyclic guanosine monophosphate (GMP) and protein kinase (Schmidt et al., 1993). NO activates soluble guanylyl cyclase and increases the intracellular levels of cyclic guanine monophosphate (cGMP) that act as effectors such as cyclic nucleotide–gated channels, protein kinases, and phosphodiesterases (Shah and MacCarthy, 2000). In neuronal tissues, NO activates neurotransmitter release, receptor efficacy, signaling, and synaptic plasticity and NO nitrosylates a variety of proteins including NMDA receptor subunits, catalytic subunits of caspases, α-tubulin, and ion-dependent ATPases (Calabrese et al., 2007). Similarly, NO can also mediate cGMP-independent actions, resulting in modulation of ion channel functions and reactive oxygen species (Davis et al., 2001). An increased NO production due to exposure to short-term stressors and its decreased production upon long-term adverse effects on the environmental system have been well-documented (Busse et al., 1995; Kumar and Chanana, 2017; Bernatova et al., 2018). Excess NO from iNOS, on the other hand, can bind with thiols to produce nitrosothiols (Smith and Marletta, 2012) and it is not still clear whether these nitrosothiols act as signaling molecules or create nitrosative stress in animals.

NO sensitizes many physiological processes that include cell proliferation and differentiation, neurotransmission, and apoptosis (Bredt and Snyder, 1992; Alderton et al., 2001; Stuart-Smith, 2004). NO is also involved in the release of hormones and immune defense (Murad, 1994). NO acts as a modulator of synaptic plasticity, releases neurotransmitters, diffuses through membranes, and affects many nearby cells (Holmqvist et al., 2004). NO has a distinct role in the regulation of neuroendocrine functions (Cioni et al., 2002) and plays a major role in cell death due to its conversion to NO to highly oxidative peroxynitrite and glutamate neurotoxicity (Dawson and Dawson, 1996). Macrophages under pathogenicity release NO as a defense molecule to protect the immune system against invading pathogens due to its ability to interfere with the DNA synthesis (Chien et al., 2004; Tripathi et al., 2007). The iNOS-mediated NO production that forms the iNOS/NO system appears to defend against pathophysiological conditions in mammals (Payne et al., 2001) and in fishes (Hasan et al., 2020; Peter and Gayathry, 2021). NO gets inactivated by superoxide anion (O_2_^−^) and forms potent oxidant peroxynitrite (ONOO^−^), which can cause S-nitrosylation of biomolecules and oxidative damage (Lee et al., 2003; Mikkelsen and Wardman, 2003). In addition, superoxide, Fe^2+^, Fe^3+^, and O_2_ scavenged by hemoglobin can quickly convert NO to nitrates or nitrites (Snyder and Bredt, 1992; Kiechle and Malinski, 1993).

### Nitric Oxide in Fish

Irrespective of the growing body of evidence for the involvement of NOS and its physiological implication in nonmammalian vertebrates, the information on the iNOS/NO system is very limited in ectothermic vertebrates, including fishes. The presence of varied isoforms of NOS has been demonstrated in many fish species, including lungfishes (Perry et al., 2016; Amelio and Garofalo, 2020). Likewise, various physiological functions of NO have also been documented in fish species. Distinct isoforms of NOS: nNOS and iNOS have been identified in adult and early developmental stages in fish tissues with the capacity to generate NO (Tota and Wang, 2005; Pietsch et al., 2008; Andreakis et al., 2011; Yao et al., 2014). Production of NO through the expression of nNOS has been documented in the gills of killifish (*Fundulus heteroclitus*) (Jennings et al., 2004; Hyndman et al., 2006) and nNOS immunoreactivity in the gills of killifish (Evans, 2002). Similarly, the presence of nNOS in the gill tissues has also been confirmed for neurons in the parenchyma beneath the gill epithelium of some catfish species (Zaccone et al., 2006), larvae and juvenile trout (Gallo and Civinini, 2001), and Nile tilapia, *Oreochromis niloticus* (Bordieri et al., 2005). Likewise, nNOS-like immunoreactivity in neurons of the intestine of zebrafish, *Danio rerio* (Holmberg et al., 2006) and the presence of iNOS in the head kidney and liver of rainbow trout (*Oncorhynchus mykiss*) had been reported (Barroso et al., 2000).

A proper modulation of cardiac function is crucial for stress response as it allows blood perfusion to the whole organism, particularly to respiratory organs and the brain. The major signaling pathways in cardiac cells that are activated for maintaining molecular equilibrium improve the capacity of stress tolerance in fish (Filice et al., 2021). Modulation in cardiovascular activity is, thus, directly linked to almost all aspects of the physiological response to internal or external challenges (Farrell et al., 2009; Farrell and Smith, 2017). Consequently, the pumping capacity of the heart that sensitizes both cellular and systemic ionic gradients regulates blood pressure and the performance of osmoregulatory organs, including the gills and kidneys (Olson, 1997). The neuroendocrine and autocrine/paracrine signaling molecules that target the osmotic epithelia direct ionic homeostasis *via* modulating the functions of ion transporter proteins including Na^+^/K^+^-ATPase (McCormick, 2001; Marshall and Grosell, 2006; Perry et al., 2016; Peter and Gayathry, 2021). In fishes, the NO/NOS system is fundamental as it modulates the basal cardiac performance and is involved in the control of many adaptive responses to stress, including those related to variations in O_2_ and thermal regimes (Carnevale et al., 2021; Filice et al., 2021).

Tissue-specific response of the NOS/NO system that tunes with organ readjustment operates in lungfish, *Prototerus annectens*, during induced estivation and arousal (Garofalo et al., 2015). For example, in these fish, mRNA expression of HIF-1∝ showed an inverse correlation with eNOS expression. Likewise, in *Prototerus dolloi,* an obligatory air-breather, the eNOS in the heart and kidneys appear as the major isoform of NOS with respect to iNOS or nNOS located in the epicardium (Amelio et al., 2008). Similarly, a critical role of the nNOS/NO system in shoaling behavior has been observed in zebrafish (Penglee et al., 2021). In addition, a role for the eNOS/NO system in morpho-functional readjustment in the cardiac and skeletal muscle has been presented in African lungfish (Amelio et al., 2020). They observed that the NOS/NO system is crucial in signaling transduction/integration networks during environmental challenges such as temperature, dehydration, and inactivity right from the onset, during estivation, and arousal for entering freshwater life (Amelio et al., 2020). The presence of the NOS/NO system in African lungfish, thus, presents phylogenetic roots of NO biosynthesis (Andreakis et al., 2011; Amelio et al., 2013; 2020).

### Transepithelial Transport in Fish

The gills are the primary corridor for molecular exchange between the internal milieu of a fish and its environment. Apart from respiratory gas exchanges, water and ions can quite readily cross this integumental barrier. The complex epithelia of this multifunctional organ possess at least seven types of cells, namely, mucus cells, chloride cells (CCs), pavement cells, respiratory cells, basal layer cells, undifferentiated cells, and neuroepithelial cells (Evans et al., 1999; Peter, 2007). The CCs, also known as ionocytes or mitochondria-rich cells, comprise up to 10% of the total number of branchial epithelium cells and actively participate in ion transport. In freshwater gills, CCs are involved in the absorption of ions (Perry, 1997), while in seawater, they are mainly concerned with the secretion of ions (Marshall, 2002; Evans et al., 2005). In a strongly hyposmotic medium such as freshwater, the gills are quite resistant to the permeation of water. Nevertheless, the high osmotic gradients across the gills cause a net osmotic inflow of water, which is eliminated through the kidneys. The necessary salts are obtained only partly through food (Bentley, 1998). It has become clear that the branchial epithelium is under the control of a variety of endocrine and paracrine factors that play a role in controlling its permeability and ionic transport (Olson, 1997; Peter et al., 2000, 2011; Evans et al., 2005; Peter and Peter, 2011; Guh et al., 2015).

The kidneys and intestines play important roles in ion regulation in fishes of both freshwater and seawater, though their function is entirely different under both conditions (Katz, 1982; Marshall and Grosell, 2006). Facing the problem of osmotic water load and ion loss, the primary function of freshwater teleost kidneys is to excrete excess water, while reabsorbing most of the filtered solutes. The glomerular filtration rate of these fishes is higher than that of marine teleosts, and as much as 95% of the filtered water can be excreted as dilute urine, which implies that the renal tubules of freshwater teleosts must have low water permeability. In contrast to the kidneys of reptiles, birds, and mammals, teleost kidney exhibits neither zonation, such as the cortex and medulla, nor a countercurrent system of tubular and vascular elements. Consequently, teleosts are unable to excrete hyperosmotic urine (Bentley 1998; Walsh, 1998; Islam et al., 2020).

In the gills, Na^+^ is secreted by the paracellular pathway involving Na^+^, K^+^-ATPase (NKA) through a junction between chloride cells and adjacent accessory cells and is regulated by environmental salinity (McCormick, 1995; Marshall and Bryson, 1998; Marshall and Grosell, 2006; Hiroi and McCormick, 2012; Guh et al., 2015). NKA is the driving force for this process. This transporter is extremely abundant in ionocytes, and it is located along the complex basolateral membrane and/or the tubular system in these cells (Peter et al., 2000, 2011; Hiroi and McCormick, 2012; Dutta et al., 2019). A positive correlation between environmental salinity and the biochemical activity of branchial NKA has been reported for several teleost fishes (Nolan et., 1999; D’Cotta et al., 2000; Peter et al., 2011). There have been several studies on the physiological and structural characteristics and on the dynamics of the chloride cells in freshwater and seawater fish (Van der Heijden et al., 1999; McCormick et al., 2005; Peter, 2007; Peter et al., 2011). Furthermore, changes in structural signs of high chloride cell activity and high gill NKA activity have been reported for seawater-adapted fishes (Nolan et al., 1999). NKA is expressed abundantly in the tubular system of fish kidney tubules (Nolan et al., 1999; Peter et al., 2000; Peter and Gayathry, 2021) and intestinal epithelia (Peter, 2011; Peter and Gayathry, 2021) and performs an integral role in the regulation of homeostasis of Na^+^ and K^+^ ions.

Fishes maintain homeostasis of intra and extracellular fluid composition with the help of gills, kidneys, and intestines. An array of hormones is involved in hydromineral balance in teleosts, in particular Na^+^ homeostasis (Balment and Hazon, 1998; McCormick, 2001; Evans et al., 2005; Iwama et al., 2006; Peter, 2011). Depending on the physiological demands, many hormones are involved in the control of ion regulation either singly or in concert, again depending on ambient water conditions (Bentley, 1998). For instance, cortisol, the main product of corticosteroidogenesis in the interrenal (adrenal) cells located in the head kidney, has traditionally been associated with teleost ion regulation in marine fish (Wendelaar Bonga, 1997; McCormick, 2001). However, cortisol also plays an important role in the ion regulation of freshwater fishes as is evident from the fact that cortisol 1) affects monovalent ion exchange in several species of freshwater fish (Dang et al., 2000), 2) increases NKA levels in the gut and gills of freshwater eels (Marshall and Grosell, 2006), 3) promotes branchial calcium uptake in freshwater rainbow trout (Flik and Perry, 1989), and 4) increases the numbers of ionocytes in freshwater tilapia (Dang et al., 2000). Furthermore, a positive correlation of branchial chloride cell density with plasma cortisol levels (Dang et al., 2000) and modification of NKA activities in the gills, kidneys, and intestines by cortisol (Nolan et al., 1999) have been demonstrated in tilapia.

### Fish Ionocytes

As a multifunctional organ, fish gills show dramatic functional changes in response to stressors. Fish gills possess varied subtypes of brachial ionocytes as the major ion transporting cells that are responsible for transepithelial ion transport (Dymowska et al., 2012). Freshwater fishes passively lose ions to the more dilute environment across the gills. The active uptake of Na^+^ and Cl^−^ occurring in the gills is the main compensation for maintaining homeostasis in freshwater teleosts. In the FW fish gills, passive Na^+^ channels, apical electroneutral Na^+^/H^+^ exchangers (NHEs), and NKCC are involved in Na^+^ transport (Marshall and Grosell, 2006). Basolaterally localized NKA, an active Na^+^ transporter, provides electromotive force for the apical entry of Na^+^ into the cell, which also depends on the apical vH^+^ATPase which is electrochemically coupled to NKA (Grosell and Wood, 2002). Uptake of Cl^−^ across the freshwater fish gill also takes place *via* an apical anion exchanger, Cl^-^/HCO_3_^-^, functionally linked to carbonic anhydrase and basolateral vH^+^ATPase (Dymowska et al., 2012). The basolateral vH^+^ATPase provides the necessary driving force to overcome the unfavorable gradient for Cl^−^ uptake. In addition, PMCA and Na^+^/Ca^2+^ exchanger (NCE) that are located on the basolateral membrane also play an important role in the exchange of Na^+^ against Ca^2+^ (Brini and Carafoli, 2011; Dymowska et al., 2012).

The kidneys of freshwater fishes excrete excess water and reabsorb ions from the glomerular filtrate. The nephron of freshwater teleosts comprises a well-developed glomerulus, proximal tubule, distal tubule, and collecting tubule (Marshall and Grosell, 2006). Na^+^ uptake in renal tubules is accompanied by extrusion of an equal amount of acid equivalents through NHE (Marshall and Bryson, 1991). Structurally distinct NKA-immunostained discrete regions of the renal tubules have been found in the climbing perch that appears to be sensitive to the iNOS/NO system (Peter and Gayathry, 2021). The single layer of columnar epithelial cells in the proximal tubules is equipped with a PAS-positive brush border at the apical membrane, whereas a single layer of cuboidal cells with a centrally located nucleus that lacked an apical brush border was found in the distal renal tubular segment. On the contrary, the collecting renal tubule had a single layer of columnar epithelial cells with a centrally located nucleus (Peter and Gayathry, 2021). The specific NKA-immunoreactivity that responds to the iNOS/NO system in these morphologically distinct ionocytes distributed in proximal, distal, and collecting tubules suggests that the iNOS/NO system has the capacity to direct the Na^+^ transport in these varied renal ionocytes of perch (Peter and Gayathry, 2021).

The renal tubules in fish kidney are equipped to reabsorb Na^+^ with active hydrolysis of NKA localized in the basolateral membrane (Tipsmark and Madsen, 2003). Regulation of NKA by the NO-cGMP messenger system in the kidney of brown trout, *Salmo trutta*, has been documented (Tipsmark and Madsen, 2003). Localization of nNOS in kidney tubules has been reported in rainbow trout (Jimenez et al., 2001). A modulatory action of NO donor, SNP on NKA regulation in osmoregulatory epithelia and its potential regulatory role in acid–base regulation during confinement stress has also been documented in air-breathing fish, *Anabas testudineus* (Peter, 2013b). A similar regulatory role of NO has been documented in fishes, especially in the cardiovascular system (Hansen and Jensen, 2010), the homeostatic functions of the gill apparatus (Jensen, 2007), acid–base regulation (Peter, 2013), protective role against hypoxia stress (Fago and Jensen, 2015; Gattuso et al., 2018), mitochondrial respiratory control, and reproductive function (Jensen, 2007; Tripathi and Krishna, 2008; Nath et al., 2019).

The intestinal epithelia of FW fish perform absorption of essential nutrients along with ions including Na^+^ and Cl^-^ from the intestinal lumen for replacing salt loss by diffusion to the dilute environment (Grosell, 2006; Taylor and Grosell, 2006). Transepithelial transport of these ions across the intestinal epithelia ultimately depends on the basolateral membrane–bound NKA, which establishes a transmembrane electrochemical gradient for Na^+^ (Figure 2). The salt absorption begins with the coupled entry of Na^+^ and Cl^−^ through NKCC (Hiroi et al., 2008). Na^+^ enters the cell and is subsequently extruded by the NKA, whereas Cl^−^ exists either passively across the basolateral membrane or through the K^+^/Cl^−^ symport (KCS) (Movileanu et al., 1998).

## Na^+^/K^+^-ATpase

The high extracellular Na^+^ and high intracellular K^+^ together with concurrent low extracellular K^+^ and low intracellular Na^+^ in almost all animal cells create an ion gradient across the plasma membrane. The resting potential of a typical cell is −70 mV, and K^+^ tends to flow out of the cell, as its equilibrium potential (−91 mV) is more negative than the transmembrane potential. Sodium ions have a very strong force driving them into the cell since both the electrical and chemical gradients favor Na^+^ uptake. The sodium pump; Na^+^/K^+^-ATPase (NKA), is necessary for proper cellular function as it preserves the ionic gradients across the cell membrane, and thus the membrane potential and osmotic equilibrium of the cell (Skou and Esmann, 1992). This hydrolase enzyme pumps 3 Na^+^ and 2 K^+^ ions against their concentration gradient at the expense of an ATP molecule (Skou and Esmann, 1992; Forrest, 2014). This transport through the sodium pump maintains transmembrane gradients for the ions and produces a convenient driving force for the secondary transport of metabolic substrates such as amino acids and glucose. The nonequivalent transport is electrogenic and leads to the generation of a transmembrane electrical potential, allowing cells to become excitable. NKA, a P-type ion-dependent ATPase, contains a transmembrane catalytic *α* subunit, which contains an ATP-binding site and usually a smaller *β* subunit, which may have regulatory functions (Kaplan, 2002). During the transport process, at least one of the *α* subunits is phosphorylated, and then transported ions are thought to move through the phosphorylated subunit.

The sodium pump molecule is a hetero-oligomer and consists of three subunits *α*, *β,* and *γ* (FXYD protein), in which *α* and *β* are necessary for ion pumping and dimerize to form functional NKA, while the third subunit regulates the pumping function (Kaplan, 2002; Jorgensen et al., 2003; Garty and Karlish, 2006). The catalytic α-subunit is a large protein of 110 kDa, containing binding sites for ions, ATP, and ouabain and a phosphorylation site (Skou and Esmann, 1992; Therien and Blostein, 2000; Laursen et al., 2013), and the β-subunit interacts with the α-subunit and is involved in regulating ion-binding affinity of the enzyme complex (Blanco and Mercer, 1998). Cellular regulation of pump expression can be controlled by the rate of synthesis of the pump subunits and delivery to the membrane. The environmental and hormonal factors can increase the sodium pump activity per cell by mainly three mechanisms: by increasing the turnover of the pumps that are already present in the membrane, through the insertion of more pumps into the cell membrane, and by increasing the transcription or translation of pump subunits, resulting in increased pump sites in the membrane. NKA is known to be the receptor for the cardiac glycoside family, which includes ouabain and digoxin, and is specifically inhibited upon binding with these substances (Lingrel et al., 1994). The function and activity of NKA have been characterized and have been shown to be essential to excretory and osmoregulatory functions. NKA is among the integral membrane protein that is sensitive to variation in membrane physical properties (Hulbert and Else, 1999). The functionality of NKA in the osmoregulatory epithelia that drive systemic Na^+^ homeostasis makes it an index of osmoregulatory performance in fishes (Dang et al., 2000).

### Inducible Nitric Oxide Synthase/Nitric Oxide System and Na^+^/K^+^-ATPase Regulation

**FIGURE 3:**
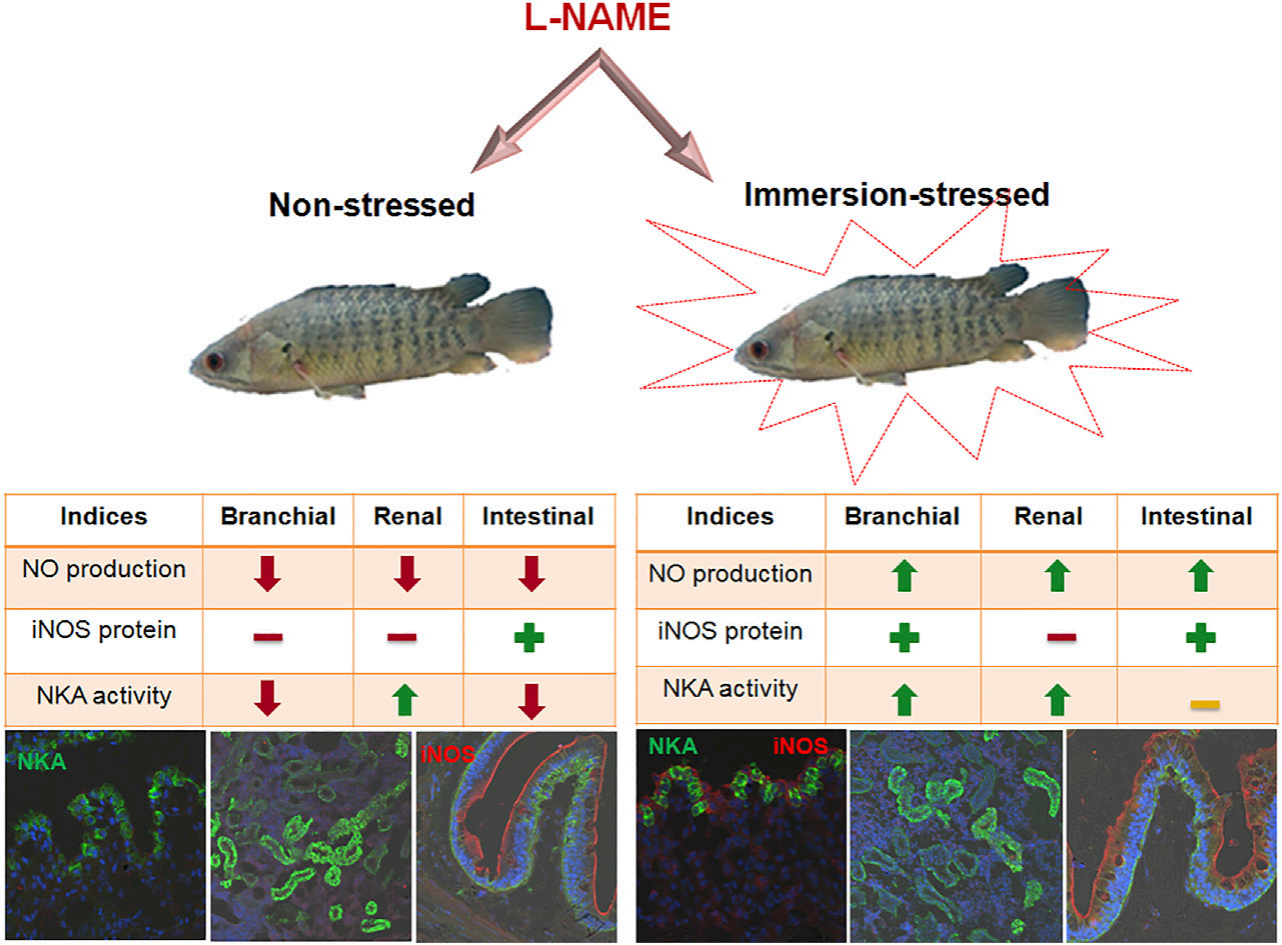
Schematic chart showing the *in vivo* action of L-NAME on NKA dynamics and the iNOS/NO system in ionocytes of air-breathing fish *A. testudineus* under hypoxemia stress. Treatment of the nitric oxide synthase inhibitor, L-NAME, in non-stressed fish suppressed the NO release in the branchial, renal, and intestinal epithelia of this fish. But, L-NAME challenge in hypoxemia-stressed fish delivered more endogenous NO production in epithelial ionocytes due to the rise in inducible nitric oxide synthase (iNOS) protein abundance, especially in branchial and intestinal epithelia. This activation of the iNOS/NO system in hypoxic fish demands multidimensional regulation of NKA in these ionocytes This resistance response of the iNOS/NO system further allows the air-breathing fish to restore the compromised epithelial Na^+^ transport and defend against the life-threatening hypoxemia condition (adopted from Peter and Gayathry, 2021).

Modulation of Na^+^ homeostatic control during hypoxia tolerance has been presented in the gill-breathing fishes (Malyshev and Manukhina, 1998; Ebbesson et al., 2005; Gerber et al., 2019). Recently, a role for the iNOS/NO system in NKA-driven epithelial Na^+^ transport has been documented in *A. testudineus*, an obligate air-breathing fish that shows resistance to acute hypoxemia (Peter and Gayathry, 2021). In these hypoxemic fish, treatment of L-NAME elevated the iNOS protein abundance and subsequent endogenous NO production, which is correlated with a rise in NKA activity that occurred in the branchial epithelia that showed a lowered NKA activity in hypoxemic condition (Peter and Gayathry, 2021). They have also demonstrated that activation of the iNOS/NO system in ionocyte epithelia of hypoxic fish modulated the NKAα-subunit protein abundance and transcript abundance of Nkaα1 subunit isoforms and that yielded compelling evidence for multidimensional regulation of NKA (Figure 3).

A tight regulation of NO availability occurs due to the concerted actions of NOS enzymes and the metabolites of NO, including nitrate and nitrite (Hansen and Jensen, 2010). Reduction of the endogenous NO level has been found in osmoregulatory tissues and serum of perch after L-NAME treatment that produced inhibition of the iNOS protein (Peter and Gayathry, 2021). A similar report on the inhibitory action of L-NAME on NO production has been reported in many gill-breathing fish species in their non-stressed condition (Ebbesson et al., 2005; McNeill and Perry, 2006). Isoforms of nNOS and iNOS produce endogenous NO and have been identified in the branchial epithelia of fish (Ebbesson et al., 2005). Similarly, higher expression of the nNOS and iNOS protein has also been identified in the gills (Ebbesson et al., 2005; Hyndman et al., 2006), kidneys (Jimenez et al., 2001), and gut (Olsson and Holmgren, 1997) of gill-breathing fishes. The activation of the iNOS/NO system in stressed *A. testudineus* further points to its adaptive value in counteracting acute hypoxemia (Peter and Gayathry, 2021). A similar rise in NO production due to activation of iNOS expression has been demonstrated in hypoxic rainbow trout (Gerber et al., 2016, 2017). In addition, hypoxia has been shown to activate iNOS expression in brown trout (Jensen et al., 2015). Moreover, a protective role of iNOS against oxidative stress and ammonia tolerance has been reported in the hepatocytes of Magur catfish (Hasan et al., 2020). Similarly, higher iNOS protein abundance due to L-NAME exposure in the mucosal layer and rodlet cells in intestinal villi has been observed in hypoxemic perch, which further suggests an activated immune response of the immunity-delivering cells in these fish (Peter and Gayathry, 2021). Delivery of NO by iNOS has been shown in mucosal surface/brush border of the epithelial layer and rodlet cells as these cells act as the first line of defense against pathogens (Parra et al., 2015) in the site for innate immune response (Manera and Dezfuli, 2004; Reite, 2005; Reite and Evensen, 2006) in the intestines of carp and eel (Bosi et al., 2018). Considering the participatory role of the iNOS/NO system in the osmoregulatory epithelia of perch in response to hypoxemia further suggests a lead role for this molecular signal in renal epithelia with respect to its Na^+^ handling performance against the branchial and intestinal epithelia (Peter and Gayathry, 2021; Figure 3). Interestingly, a similar pattern of osmotic integration after SNP challenge has been found in this fish where inhibition of brachial NKA activity prevails (Peter, 2013) as in other teleosts (Tipsmark, 2003).

Branchial ionocytes in freshwater teleosts absorb Na^+^ and Cl^−^ from water by utilizing apical transporters such as NHE, H^+^-ATPase, and NKCC and actively pumps out Na^+^ *via* NKA into the blood (Evans et al., 2005; McCormick et al., 2009). These ionocytes with their rich NKA serve as a sensitive target to environmental stressors and to many stress-responsive hormones such as cortisol and stress modified hormone like thyroid hormone (Peter, 2011; Peter and Peter, 2011; Tipsmark et al., 2011). A switching of Nkaα1 isoforms that demands differential regulation and contributes to spatial and preferential regulation among the varied epithelia has been observed and is critical during the activation of the iNOS/NO system specifically to defend against acute hypoxia. A genomic response of the iNOS/NO system to Nkaα1 subunit isoform regulation in hypoxemic climbing perch has been documented, where it could reshuffle its isoform combination and finally restore the rate of Na^+^ transport (Peter and Gayathry, 2021). This agrees with the differential kinetic properties of the α1 subunit isoforms of NKA, which are critical for its physiological functions and regulation, including the binding of Na^+^ or K^+^ ions and ATP in ionocytes (McCormick et al., 2009). In addition, a vital role of NO in NKA protein availability has been demonstrated in trout gills and kidneys by Tipsmark and Madsen (2003). Interestingly, activation of the iNOS/NO system that promotes the transcriptional activation of Nkaα1b in the renal epithelia and its deactivation in the branchial and intestinal epithelia of hypoxic fish further suggest a lead role of Nkaα1b isoform during hypoxemia resistance in this fish (Peter and Gayathry, 2021).

Similar activation of iNOS and NKA proteins has been observed in the gills of anadromous salmon, *S. salar* (Ebbesson et al., 2005). Partial inhibition of NKA activity by the NO donor has been observed in salmon gills and the gills and kidneys of brown trout (Tipsmark and Madsen, 2003). They also found a rise in whole-tissue cGMP concentration that inhibited NKA activity after treatment of SNP, an NO donor, in brown trout (Tipsmark and Madsen, 2003). Likewise, nNOS is involved in the transport of Na^+^ and Cl^−^ in the opercular epithelium and gill cells, and inhibition of gill NKA after NO has been observed in these seawater-acclimatized Atlantic salmon (Ebbesson et al., 2005). In addition, the correlation between nNOS and NKA has been demonstrated in the opercular skin of killifish, suggesting that the transport of Na^+^ and Cl^−^ across the opercular skin is under endogenously produced NO (Evans, 2002). These studies have suggested that NO can act as a regulator of ion transport, particularly Na^+^ transport, in fish gills.

### Inducible Nitric Oxide Synthase/Nitric Oxide System and Stress and Ease Response in Fish

**FIGURE 4:**
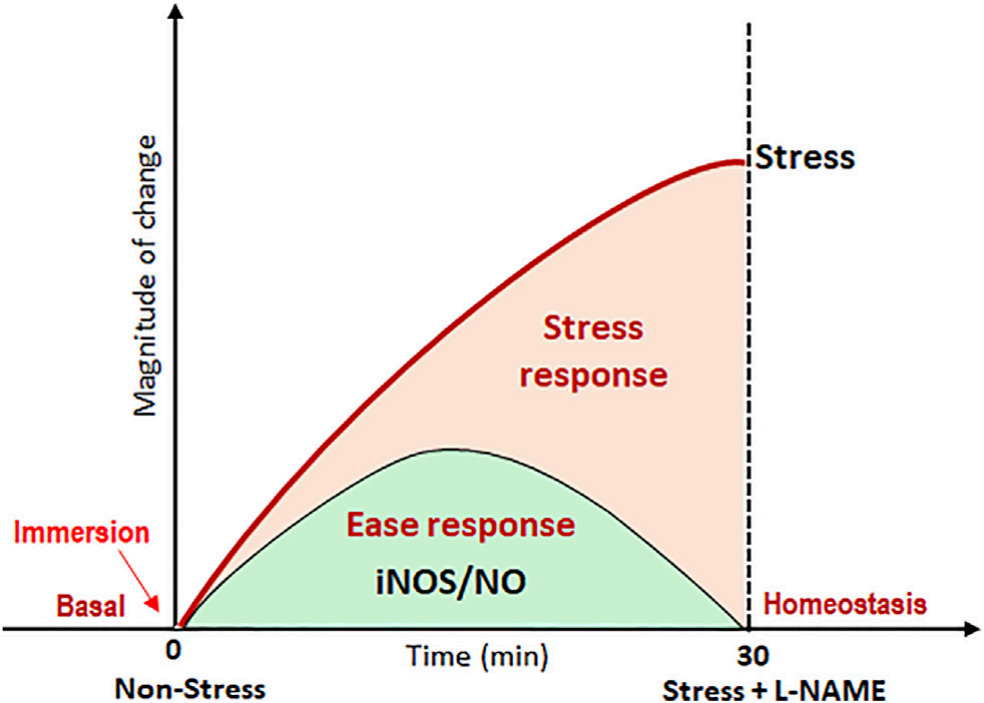
Typical homeostatic pattern of stress response involves NKA-driven Na^+^ homeostasis and ease response. Here, the response of NKA function was plotted when the fish was subjected to 30 min immersion stress against the nonstressed condition. The highest magnitude of ionosmotic stress response is represented as the highest peak in the parabola when the fish were held at a 30-min time frame. L-NAME–induced activation of the iNOS/NO system in the ionocytes of stressed fish initiated a recovery response by lowering the magnitude of NKA-driven Na^+^ transport. This ability of the iNOS/NO system to attenuate the hypoxemia-induced ionosmotic stress response depicts a major role of the iNOS/NO system as a biomarker for stress and ease response in fish.

The iNOS/NO system appears to be involved in many stressed fishes (Table 1). For example, activation of iNOS/NO can be found in the *Clarius magur* and *Heteropnestes fossilis* after hyper-ammonia stress, water shortage stress, and UV-B radiation (Choudhury and Saha, 2012a, 2012b; Kumari et al., 2018; Hasan et al., 2020; Das et al., 2021). It is plausible to ascertain that fish could rely on the iNOS/NO system to regain the basal homeostatic state through recovery activities. It appears that iNOS/NO that gets activated in hypoxic fish could favor the process of stress acclimation to exhibit the cycle of adaptive response (Figure 1). It is evident that in the cycle of the adaptive response of fish, induction of stress response occurs as a result of hypoxia, which is followed by the induction of ease response due to the activation of the iNOS/NO system (Figure 1; 4). Consequently, the phases of stress and ease in the adaptive response cycle reflect the physiological adaptive status of fish to conditions of stress and recovery. As a physiological correction process, ease response can promote recovery activity in fish against stress condition (Peter, 2013). The hypoxia-induced alterations in Na^+^ transport activity in ionocytes have been found corrected by the activation of the iNOS/NO system in the air-breathing perch (Peter and Gayathry, 2021). This recovery response clearly provides evidence for the iNOS/NO system as a molecular signal that could mediate a basal Na^+^ homeostatic control by rectifying the disturbed Na^+^ transport activity (Figure 4). Apparently, the iNOS/NO system can, thus, act as an ease signal or a promoter of ease response. Considering the activation ability of the iNOS/NO system during stressor exposure and its ability to attenuate the magnitude of stress response in fish, particularly during NKA-driven Na^+^ homeostasis, it is evident that direct control of the iNOS/NO system exists during the adaptive mechanism in fish. This has led to considering the iNOS/NO system as a biomarker for stress and ease response in fish.

**TABLE 1:** Action of NO by the various isoforms of NOS on different tissues of fish after treatment with various NO agonists and NOS inhibitors.

Condition/agonist/inhibitor	Target	Variable	Tissue	Fish species	References
SNP and PAPA- NONOate		NKA	Gills; kidney	*Salmo trutta*	Tipsmark and Madsen, (2003)
SNP and PAPA- NONOate	eNOS, nNOS, iNOS	NKA	Gills	*Salmo salar*	Ebbesson et al., (2005)
SNP+ immersion stress		NKA	Gills, kidney, and intestine	*Anabas testudineus*	Peter, (2013)
High external ammonia	iNOS		Gills; kidney	*Heteropneustes fossilis*	Choudhury and Saha, (2012a)
Water shortage stress	iNOS		Gills; kidney	*Heteropneustes fossilis*	Choudhury and Saha, (2012b)
lipopolysaccharide (LPS)	iNOS		Liver	*Heteropneustes fossilis*	Choudhury and Saha, (2016)
Hyper-ammonia stress	iNOS		Gills; kidney	*Clarius magur*	Kumari et al. (2018)
Salinity	iNOS nNOS		Gills; kidney	*Onchorhynchus mykiss*	Gerber et al. (2016)
High environmental ammonia	iNOS		Primary hepatocytes	*Clarias magur*	Hasan et al. (2020)
L-NAME + Hypoxemia	iNOS	NKA	Gills, kidney, and intestine	*Anabas testudineus*	Peter and Gayathry, (2021)
UV-B radiation	iNOS		Gills, kidney, and intestine	*Heteropneustes fossilis*	Das et al. (2021)

### Future Perspectives

The mechanistic understanding of the iNOS/NO system in osmotic epithelia is limited in fishes. For example, the molecular mechanisms of the iNOS/NO system that underlies ion transporter function in cardiomyocytes and hepatocytes are to be delineated for identifying the therapeutic potential of the iNOS/NO system. Similarly, the integrative action of the iNOS/NO system on the ion transport capacity of ionocytes needs to be investigated while considering the involvement of various active transporters and ion channels, including NKCC, NHE, NHS, vATPase, and ClC2. Moreover, it seems to be relevant for delineating the energetics of the iNOS/NO system in the osmotic potential for maintaining cell populations. Likewise, the molecular mechanism of cell signaling involving GC-mediated or SAC-driven cell signaling is to be elucidated as these potential lines of investigation can yield a better understanding of the integrative signaling of iNOS/NO with various cell signaling machineries and different pathways. Furthermore, it is also important to find out how iNOS/NO interacts with the receptors of neurotransmitters and neurohormones. It appears that the iNOS/NO system can act as a marker of stress and ease response due to its fundamental role in the regulation of NKA-driven cellular and systemic Na^+^ homeostasis in the vertebrate model.
